# Effect of Camel Peptide on the Biofilm of *Staphylococcus epidermidis* and *Staphylococcus haemolyticus* Formed on Orthopedic Implants

**DOI:** 10.3390/antibiotics12121671

**Published:** 2023-11-28

**Authors:** Joanna Nowicka, Adriana Janczura, Magdalena Pajączkowska, Grzegorz Chodaczek, Patrycja Szymczyk-Ziółkowska, Urszula Walczuk, Grażyna Gościniak

**Affiliations:** 1 Department of Microbiology, Faculty of Medicine, Medical University, 50-368 Wrocław, Poland; magdalena.pajaczkowska@umw.edu.pl (M.P.); urszula.walczuk@umw.edu.pl (U.W.); grazyna.gosciniak@umw.edu.pl (G.G.); 2 Bioimaging Laboratory, Łukasiewicz Research Network—PORT Polish Center for Technology Development, 54-066 Wrocław, Poland; grzegorz.chodaczek@port.lukasiewicz.gov.pl; 3 Centre for Advanced Manufacturing Technologies (CAMT/FPC), Faculty of Mechanical Engineering, Wroclaw University of Science and Technology, Łukasiewicza 5, 50-371 Wroclaw, Poland; patrycja.e.szymczyk@pwr.edu.pl

**Keywords:** Camel peptide, CNS, biofilm, steel implants

## Abstract

The increasing bacterial drug resistance and the associated challenges in the treatment of infections warrant the search for alternative therapeutic methods. Hope is placed in antimicrobial peptides, which have a broad spectrum of action and are effective against strains which are resistant to conventional antibiotics. Antimicrobial peptides are also tested for their efficacy in the treatment of infections associated with the formation of biofilm. The aim of the present study was to examine the effect of Camel peptide on *S. epidermidis* and *S. haemolyticus* adhesion to and formation of biofilm on steel cortical bone screws and also on the process of reducing mature biofilm in orthopedic implants. The tests were performed on steel implants for osteosynthesis. The MIC value and MBEC values of the peptide were determined using the microdilution method in microtiter plates. The effect of the peptide on adhesion and biofilm formation, as well as on the activity on the preformed biofilm, was evaluated using quantitative methods and confocal microscopy. The presented research results indicate that the peptide exhibits very good antimicrobial properties against the analyzed strains. Concentrations above MIC reduced biofilm in the range of 90–99%.

## 1. Introduction

The use of various types of biomaterials is the basis of treatment in orthopedic, trauma–orthopedic and trauma surgery departments. The implantation of biomaterials can improve the quality of life of patients, but unfortunately, it also carries the risk of infectious complications. Coagulase-negative staphylococci (CNS) are the etiological factor in 20–40% of this type of infections [1,2,3].

Coagulase-negative staphylococci are microorganisms that are widely spread in the environment. Although they form part of the human microbiota, mainly the skin, they are at the same time an important etiological factor of infections [4,5,6].

Infections caused by CNS, mainly by *Staphylococcus epidermidis* and *Staphylococcus haemolyticus*, can be very severe and can be a direct threat to the patient’s life. They include primarily infections connected with the implantation of a foreign object (biomaterial) into the human body. Both *Staphylococcus epidermidis* and *Staphylococcus haemolyticus* are characterized by increased adherence to biomaterials used in medicine. Their exceptional adhesive capacity can lead to the formation of biofilm. Infections associated with the formation of biofilm are known to be long-lasting, recurrent and difficult to treat [4,7,8,9,10]. This is due to biofilm resistance to antimicrobial compounds and to the elements of the human immune system [2,11].

Staphylococcal multidrug resistance is an additional factor increasing the risk of prolonged infections caused by them [4,8]. Frequently resistant to most antibiotic groups, these microorganisms are alert pathogens and are a major problem in the treatment of infections. In hospital settings, the most serious threat is posed by methicillin-resistant strains, which, in addition to showing resistance to β-lactams, are also resistant to other classes of antibiotics [4,8,12,13].

The fact that staphylococci form biofilm and have developed significant drug resistance is a challenge in treating infections caused by them and the reason why the search for alternative therapeutic methods is so crucial. Much hope with respect to the above is vested in AMPs (antimicrobial peptides), which may also become an effective weapon in fighting staphylococcal biofilm [14,15,16].

AMPs are a group of naturally occurring compounds. There are more than 3350 of them described in The Antimicrobial Peptide Database [17]. Produced both by prokaryotic and eukaryotic organisms, they are key components of the immune system of eukaryotic cells and are considered to be the most ancient mechanisms of immunity [14,18]. In humans, endogenous peptide antibiotics are the first line of defense on the skin and mucous membranes. They are found in saliva and are mainly produced by the keratinocytes in the stratum corneum, neutrophils or sweat glands [19,20]. Their increased secretion is observed in the inflammation area caused by infection [19,20]. They are short-chain proteins consisting of 12–50 amino acid residues with a molecular weight of 3–10 kDa and positive charge, which is associated with the fact that acidic amino acids significantly outnumber basic amino acids in their structure [21,22,23,24]. Their spectrum includes bacteria (both Gram-positive and Gram-negative), viruses, fungi and protozoa. They also demonstrate anticancer activity and immune-system-modulating activity. They hinder tissue destruction under the influence of the microorganisms’ enzymes, and they accelerate wound healing [20,25,26].

A majority of peptides act in a “corkscrew” fashion by penetrating bacterial cell walls, destroying them and causing cell lysis. They may also stop DNA replication and protein expression, as well as the depletion of adenosine triphosphate [26,27,28]. In Gram-positive bacteria, they are attracted to the anion groups present in peptidoglycan, while in Gram-negative bacteria, they bind to the lipopolysaccharides in the outer cell wall [29].

The first antimicrobial peptides (magainines) were discovered in the 1980s in frogs. In the above amphibians, the peptides protect the animal’s skin against microorganisms, including, in particular, water-borne bacteria [30,31]. Currently, many peptides are isolated from insects, mainly bees, in which they form part of the insects’ immune system [32,33]. Databases gathering information on peptides, their structure, properties and scope of activity or functions enable the production of new synthetic peptides [17,34,35]. Among such peptides is the Camel peptide studied in the present paper.

Camel peptide is an analog of peptides occurring naturally in the environment. It is composed of melittin and cecropin A (CA(1–7)M(2–9)) [36,37,38]. It does not possess hemolytic properties and destroys the bacterial cell wall. As it also destroys the mitochondrial membrane, it demonstrates anticancer activity [37,38]. The application of Camel peptide has been observed to inhibit the growth of mouse melanoma [38]. The analysis of the peptide’s activity against reference strains, both Gram-positive and Gram-negative, has demonstrated its good antimicrobial efficacy [28,39].

There has been promising research on the possibility of using antimicrobial peptides in microbiology and in the treatment of infections caused by various microorganisms. Especially promising is the fact that there are studies confirming their efficacy against strains that are frequently resistant to conventional antibiotics. The possibility of using peptides in infection treatment is still being investigated in both in vivo and in vitro tests. Some AMPs are at the stage of clinical and preclinical testing [14,28,39,40,41]. Peptides are tested for possible application in sepsis patients, and good results have been obtained for combination therapies involving conventional antibiotics and peptides [42]. The effect of AMPs on *Candida* biofilm has also been analyzed [43]. The analyses conducted give hope for their future application in monotherapy, as well as in combination therapy [44].

The aim of the present study was to examine the effect of Camel peptide on *S. epidermidis* and *S. haemolyticus* adhesion to and formation of biofilm on steel cortical bone screws and also on the process of reducing mature biofilm in orthopedic implants.

## 2. Results

### 2.1. Methicillin Resistance

Of the 121 strains analyzed, 38% were methicillin-sensitive and 62% were methicillin-resistant strains. Among *S. epidermidis* and *S. haemolyticus* strains, methicillin resistance was found in 36% and 88% of the strains, respectively (Table 1).

Methicillin resistance was more common in *S. haemolyticus* strains, but statistical analysis did not confirm the significance of the difference (Fisher’s test *p* = 0.068).

Detailed data regarding methicillin resistance among *S. epidermidis* and *S. haemolyticus* strains can be found in the Supplementary Material in Table S1.

### 2.2. MIC and MBEC of Camel Peptide against S. epidermidis and S. haemolyticus

**Figure 1:**
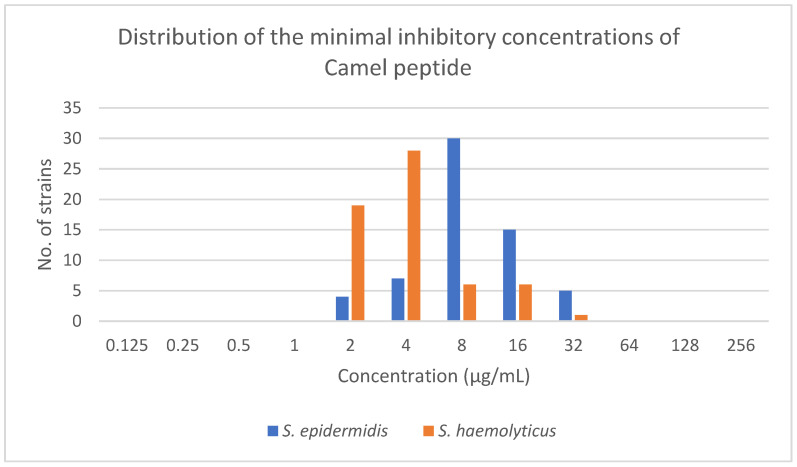
Distribution of the minimal inhibitory concentrations of Camel peptide among *S. epidermidis* and *S. haemolyticus* strains.

**Figure 2:**
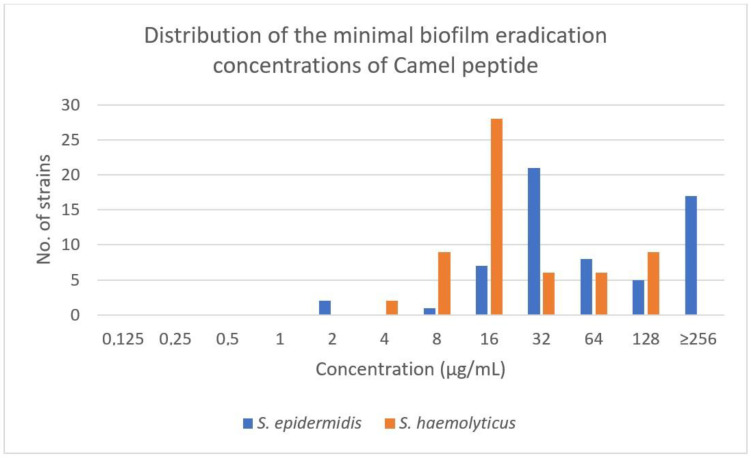
Distribution of the minimal biofilm eradication concentrations of Camel peptide among *S. epidermidis* and *S. haemolyticus* strains.

The MIC values of the Camel peptide against *S. epidermidis* ranged from 2 µg/mL to 32 µg/mL. The MBEC value ranged from 4 to >250 µg/mL (Table 2, Figure 1 and Figure 2).

In the case of *S. haemolyticus*, the range for the Camel peptide was from 2 µg/mL to 32 µg/mL and from 4 µg/mL to 128 µg/mL, respectively, for MIC and MBEC (Table 2, Figure 1 and Figure 2).

For *S. haemolyticus*, statistically significantly lower MIC values (Mann–Whitney test *p* < 0.0001) and MBEC values (Mann–Whitney test *p* < 0.0001) were demonstrated. *S. haemolyticus* strains had statistically significant lower differences between MIC and MBEC values (Mann–Whitney test *p* < 0.0001).

Detailed data on the obtained MIC values and MBEC values of Camel peptide are available in the Supplementary Material in Tables S2 and S3.

### 2.3. Effect of the Peptide on the Adhesion Ability and Biofilm Formation on Steel Cortical Bone Screws

Quantitative evaluation of the ability of all analyzed strains to form biofilm on steel cortical bone screws showed a range of cfu/mL colony-forming units from 3.2 × 10^4^ to 7.8 × 10^9^. Among *S. epidermidis* and *S. haemolyticus* strains, the range of cfu/mL units was 1.0 × 10^5^–7.8 × 10^9^ and 3.2 × 10^4^–3.4 × 10^7^, respectively.

The cfu/mL values were statistically significantly higher in the case of the *S. epidermidis* strain (Mann–Whitney test *p* < 0.0001).

Detailed data regarding quantitative evaluation (cfu/mL) of the ability to form biofilm for analyzed strains are available in the Supplementary Material in Table S4.

The analysis of the effect of the peptide (in concentrations equal to MIC, sub-MIC and above MIC) on the adhesion ability of all the investigated strains in relation to cortical bone screws showed a 95% average decrease in the number of adhered cells. The above MIC concentrations (2×MIC, 4×MIC) decreased the adhesive abilities of the staphylococci on average by 98–100%. At subinhibitory concentrations (1/2 MIC, 1/4 MIC), the number of adhered cells was lower by 60% and 70%, respectively, for concentrations of 1/2 MIC and 1/4 MIC. An increase in the number of adhered cells after the application of subinhibitory concentrations was observed in 3% of the strains. 

At concentrations equal to MIC, the biofilm formed in the presence of the peptide had on average 65% fewer cfu/mL, and for concentrations of 1/2 and 1/4 MIC, the relevant number dropped by 60% and 50%, respectively.

**Figure 3:**
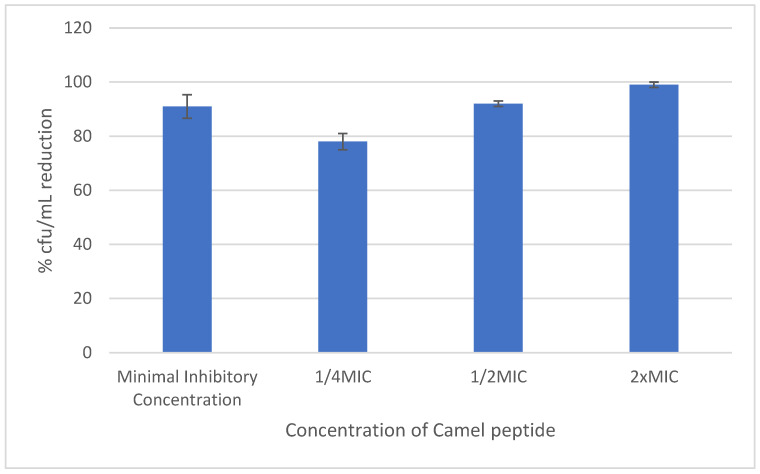
Effect of Camel peptide on the adhesion and biofilm formation of *S. haemolyticus* strain isolated from a wound.

**Figure 4:**
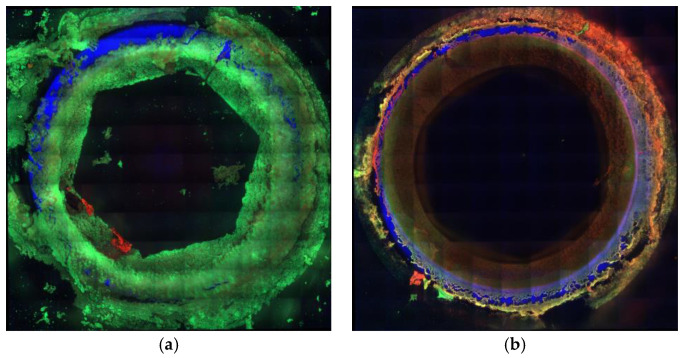
Biofilm formation in the presence of Camel peptide. Mosaic image obtained on a Zeiss Cell Observer SD confocal microscope; Steel screw; (**a**) control—without the addition of Camel peptide; (**b**) sample tested—with Camel peptide (concentration 32 µg/mL); green color—live cells; red color—dead cells; blue color—implant surface.

Figure 3 shows the effect of various concentrations of Camel peptide on biofilm formation by a selected *S. haemolyticus* strain. Figure 4 shows a confocal microscopy image and an assessment of biofilm formation in the presence of the peptide. An increase in the red signal area upon peptide treatment can be observed, indicating the presence of dead cells.

### 2.4. Effect of the Peptide on Mature Staphylococcal Biofilm

The reduction in produced biofilm caused by the peptide depended on the peptide concentration. For concentrations equal to MIC, it was 40%, while for concentrations 2× and 4×MIC, it was 90% and 99%, respectively.

**Figure 5:**
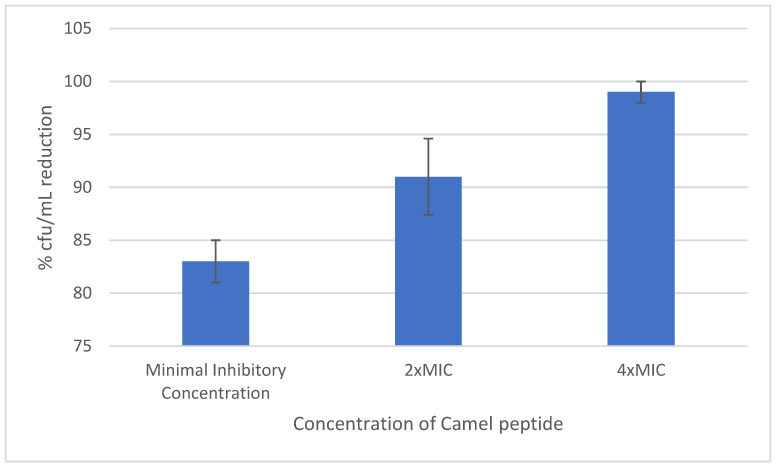
Effect of the peptide on mature staphylococcal biofilm.

Figure 5 shows the effect of various concentrations of Camel peptide on the reduction in biofilm of selected *S. haemolyticus* strains.

### 2.5. Speed of Biofilm Formation in the Presence of Camel Peptide

The analysis of the speed of biofilm formation conducted at various time intervals (at the 2nd, 4th, 8th, 24th, 48th and 72nd hour) in the presence of the peptide at concentrations equal to MIC, 1/2 MIC and 2×MIC showed that biofilm was produced more slowly in comparison with the control sample (without the presence of the peptide).

**Figure 6:**
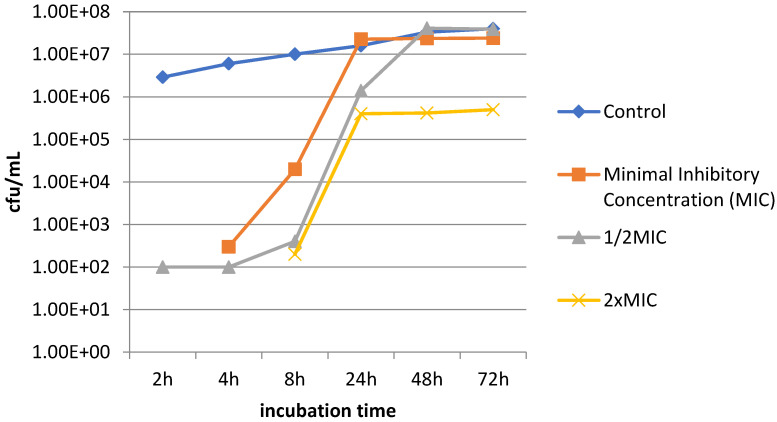
Speed of biofilm formation in the presence of various concentrations of Camel peptide. *S. epidermidis*—strain isolated from biomaterial.

Figure 6 shows a sample biofilm formation curve in the presence of various concentrations of the peptide.

## 3. Discussion

Among the possible complications after the implantation of orthopedic biomaterials is the risk of developing infections. The implanted biomaterial is susceptible to microbial colonization, which can result in the formation of biofilm structures. Biofilm can lead to tissue destruction, failure of the surgery and the need for revision surgery, as well as carrying the risk of systemic infection. Staphylococci, including *S. epidermidis* and *S. haemolyticus*, are predominant in these types of infections [45,46,47].

Apart from their ability to form biofilm, staphylococci display multidrug resistance, which is the reason why the treatment of infections caused by them is so difficult [4,8]. The methicillin resistance of staphylococci is of great significance [4,8,12,13]. Among the analyzed strains, it was much more common in the *S. haemolyticus* strains. The plasticity of the genome of this microorganism contributes to the development of its antibiotic resistance and survival in the hospital environment [46]. This species is more resistant to antibiotics than other coagulase-negative staphylococci, with clinical strains displaying a particularly broad spectrum of resistance [4]. When assessing antibiotic susceptibility among clinical strains of *S. haemolyticus*, Barros et al. showed resistance to more than three classes of antibiotics in 75% of strains. Methicillin resistance was found in 88% of the strains [48].

The creation of new compounds with antimicrobial activity as an alternative to antibiotics is of particular importance in the era of increasing bacterial drug resistance. It is also significant in the case of infections accompanied by biofilm formation. Antimicrobial peptides are a valuable group of compounds with therapeutic potential [45,49].

A comparison of both values—minimal inhibitory concentration for the planktonic form (MIC) and minimal biofilm eradication concentration (MBEC)—has shown the “higher resistance” of *S. epidermidis* and *S. haemolyticus* in biofilm form as opposed to their planktonic forms. Obviously, unlike in the case of conventional antibiotics, no clinical breakpoints for Camel peptide exist that would enable the classification of a strain as susceptible or resistant. The MBEC value of the analyzed strains for the Camel peptide increased from 2 to 32 times in relation to the MIC value, which allows the conclusion that in the produced biofilm structure, a majority of the investigated strains changed their susceptibility profile to resistant. It should be noted, however, that in the case of conventional antibiotics used in the treatment of staphylococcal infections (vancomycin, teicoplanin, daptomycin, linezolid or ciprofloxacin), the minimum concentration required for biofilm eradication may be higher than the value of minimum inhibitory concentration by as much as 10–8000 times [47,50]. Bacteria in the biofilm structure are 100 to 1000 times less sensitive than their planktonic counterparts [50].

After assessing the susceptibility of the strains to the peptide used in the study, the peptide’s effect on the adhesion process and biofilm formation on steel cortical bone screws was analyzed, as well as the peptide’s efficacy in reducing a mature biofilm structure formed on the investigated biomaterials. In both cases, the effect of the peptide was assessed in various concentrations—subinhibitory, equal to MIC and above MIC.

The evaluation of the effect of the peptide on the adhesion ability and on biofilm formation on cortical bone screws has shown a relationship between the concentration of the peptide and its ability to inhibit adhesion. The highest concentrations of the peptide eliminated the staphylococcal adhesive properties almost completely, while the lowest concentrations reduced them by 60–70%. The biofilm formed in the presence of the peptide demonstrated a reduced number of cfu/mL in all the investigated strains. The degree of mature biofilm reduction under the influence of the peptide depended on the concentration used. Concentrations above MIC reduced biofilm in the range of 90–99%.

It is worth noting that the author’s own research, including an assessment of the effect of various concentrations of the Camel peptide on the production of biofilm matrix on a polystyrene surface, has shown a reduction in the number of adhered cells but, unlike in the case of cortical bone screws, the reduction did not depend on the concentration of the peptide used. Although the lowest concentrations of the peptide produced the smallest reduction, the remaining concentrations inhibited the adhesive abilities of the strains to a similar extent. The assessment of the effect of the peptide on the biomatrix formed on the polystyrene plate situation was very similar—no dosage dependence on the degree of biofilm biomatrix eradication was demonstrated.

The analysis of the speed of biofilm formation on cortical bone screws performed as part of the next stage of the study showed a “slower” biofilm production in the presence of various concentrations of the Camel peptide in comparison with the control sample.

Antimicrobial peptides have been investigated at a number of research centers to measure their efficacy and possibilities of application in the treatment of infections with biofilm involvement [51,52,53]. Dawgul et al. showed greater activity of selected antimicrobial peptides (Palm-KK-NH_2_, Palm-RR-NH_2_, Omiganan, Pexiganan, Temporin A, Citropin 1.1) against the biofilm of Gram-positive bacteria (*Staphylococcus aureus*, *S. epidermidis*, *Streptococcus pneumoniae*, *Streptococcus pyogenes*) than against Gram-negative bacteria (*Escherichia coli*, *Pseudomonas aeruginosa*, *Proteus mirabilis*). In this case, the above activity was significantly higher compared with conventional antimicrobial compounds [54]. Palm-KK-NH_2_ and Palm-RR-NH_2_ have also been investigated for their effects on the formation and eradication of *Candida* biofilm. Peptides displayed a much higher activity than conventional antifungal agents. They were effective in inhibiting adhesion and in biofilm eradication at concentrations below and equal to MIC, while conventional antifungal agents demonstrated activity at concentrations equal to MIC or significantly above MIC [24]. It is worth noting again that subinhibitory concentrations of the Camel peptide reduced staphylococcal adhesion by 60–70%, and concentrations equal to MIC reduced mature biofilm by as much as 40%. The presented results indicate that the Camel peptide may become a valuable and effective agent in fighting bacterial biofilm, especially the biofilm occurring in orthopedic cases. It is possible that coating biomaterials or other medical devices implanted into the human body with substances including the Camel peptide would be a good direction to follow in the future to reduce the occurrence of infections associated with the use of biomaterials and involving biofilm formation. The controlled, local release of the antimicrobial peptide HHC-36 from the titanium surface was effective against both *S. aureus* and *P. aeruginosa* [55,56].

Zhao et al. [57] analyzed the activity of the Tet213 antimicrobial peptide against *S. aureus* strains isolated from orthopedic infections. The effectiveness of the analyzed peptide was assessed after 30 min and then in the 2nd, 4th, 6th and 8th hour. Using the quantitative method (measuring cfu/mL), an inhibitory effect was demonstrated on the adhesion and biofilm formation in 80% of the analyzed strains [57]. The same peptide was investigated by another group of researchers. Kazemzadeh-Narbat et al. [58] loaded the above-mentioned peptide on the surface of a microporous calcium phosphate coating and deposited it onto the surface of titanium as the drug carrier. Using the quantitative method, they demonstrated a 10-fold reduction in *S. aureus* and *P. aeruginosa* strains within 30 min from incubation of the strains with the peptide [58]. As observed by Chung, the LL-37 peptide reduces the adhesion of *P. aeruginosa* to both tissues and various types of biomaterials. The above peptide also inhibits adhesion and biofilm formation by *S. epidermidis* strains [59]. Another synthetic antibacterial peptide, ATRA1, as well as the above-mentioned natural peptide AMP LL-37 of the cathelicidin family, inhibits biofilm formation by *S. aureus* in concentrations lower than 3 µg/mL [59]. The research conducted by Gopal et al. indicates the antibacterial efficacy of peptides against the multiresistant *Acinetobacter baumanii* strain. Importantly, the investigated peptides demonstrated synergistic effects with conventional antibiotics [60]. Oppositely, in their research conducted in 2017, Bormann et al. [52] analyzed the antimicrobial activity of five short cationic antimicrobial peptides against various bacterial species (*E. coli*, *Enterococcus faecalis*, *P. aeruginosa*, *S. epidermidis*, *S. aureus*—including clinical methicilin-susceptible and methicilin-resistant isolates). The authors of the paper analyzed the effect of the peptides on bacterial biofilm, as well as their influence on osteoblast-like cells. Their results suggest high antimicrobial activity of the analyzed cationic peptides, even against bacteria within a biofilm. Using the FISH method, Bormann et al. noted a decrease in biofilm thickness and a change in its structure. Concentrations of the AMP2 peptide of 4 µg/mL and 8 µg/mL reduced the biofilm by 86% and 90%, respectively. It is also important, especially taking into account the possibility of using peptides in infection treatment, that they did not exhibit any harmful effect on human osteoblasts [52].

Jaśkiewicz et al. evaluated the activity of eight antimicrobial peptides (aurein 1.2, Camel, citropin 1.1., LL-37, omiganan, r-omiganan, pexiganan, temporin A) against the reference strain of *A. baumannii.* Among the peptides used, the lowest MIC values were shown for the Camel peptide and pexiganan. In the case of the Camel peptide, a six-fold higher MBEC value was shown at the same time [61]. When assessing the activity of the selected antimicrobial peptides (aurein 1.2, Camel, citropin 1.1, omiganan, pexiganan, temporin A)—parent compounds and their retro analogs—against Gram-positive microorganisms (*S. aureus*, *S. pneumoniae*, *E. faecalis*), Gram-negative microorganisms (*E. coli*, *Klebsiella pneumoniae*, *P. aeruginosa*) and fungi (*Aspergillus niger*, *Candida albicans*, *Candida glabrata*), Neubauer et al. showed significantly higher activity of the parent peptide. The highest activity of the Camel peptide was found against *S. pneumoniae* and against *K. pneumoniae*. r-Camel was significantly less active against the majority of microorganisms than the parent peptide [39].

## 4. Materials and Methods

### 4.1. Bacterial Strains

This study was performed on clinical coagulase-negative staphylococcus sp., including 61 *S. epidermidis* and 60 *S. haemolyticus* strains isolated from orthopedic patients from blood, wound swabs and biomaterials (screws, nails) removed from the patients’ bodies for medical indications. Clinical strains are part of the internal strain collection of the Department of Microbiology, Wroclaw Medical University.

The reference strain *S. epidermidis* RP 62A (ATCC 35984) was also included in the study.

### 4.2. Biomaterial

Osteosynthesis implants (316L stainless steel cortical bone screws; Medgal, Poland) were used in this study. The screws had a hex head, were 12 mm long and had a diameter of 4.5 mm.

### 4.3. Camel Peptide

Camel peptide (Lipopharm, Gdańsk, Poland) was used in the study.

### 4.4. Strain Storage and Culture Conditions

The analyzed strains were stored in deep freezers (−80 °C) in a liquid tryptic soy broth (TSB; Biomaxima, Lublin, Poland) medium with 15% Glycerol and cultured in Columbia agar with 5% sheep blood (Becton-Dickinson, Warszawa, Poland) at 37 °C for 18–24 h under aerobic conditions.

### 4.5. Methicillin Resistance

The resistance of the strains to methicillin was tested using the disk diffusion method (cefoxitin, 30 µg) according to EUCAST [62].

### 4.6. Minimal Inhibitory Concentration (MIC) of Camel Peptide

A suspension of McFarland standard 0.5 (Densitometer Densimat; BioMerieux, Warszawa, Poland) was prepared from an 18 h culture of the analyzed strains. The suspension was diluted 100 times and placed (100 µL each) in the relevant rows of a 96-well plate (Equimed, Wrocław, Poland). After the addition of 100 µL of Mueller Hinton broth (MHB; Biomaxima, Lublin, Poland) to each well, the geometrically diluted peptide was added (0.125 µg/mL–256 µg/mL). After incubation (24 h, 37 °C), a drop of 2,3,5-triphenyl tetrazolium chloride (TTC; Merck, Poznań, Poland) was added, incubated (2 h) and the MIC value was read. The first, highest dilution of the peptide at which no TTC reduction by live microorganisms was observed was regarded as MIC.

### 4.7. Minimal Biofilm Eradication Concentration (MBEC) of Camel Peptide

After an 18 h culture of the staphylococci, a suspension was prepared at 0.5 McFarland standard (Densitometer Densimat; BioMerieux, Warszawa, Poland), which was diluted 100 times and placed (100 µL each) in the appropriate rows of a 96-well plate (Equimed, Wrocław, Poland). Following incubation (24 h, 37 °C), the suspension was removed, the wells were rinsed 3 times with 0.9% NaCl solution and 100 µL of Mueller Hinton broth (Biomaxima, Lublin, Poland) was added. The geometrically diluted peptide (0.125 µg/mL–256 µg/mL) was placed in the respective rows of the plate and incubated, after which a drop of TTC (Merck, Poznań, Poland) solution was added. MBEC value was read after 24 h of incubation.

### 4.8. Effect of the Peptide on Adhesion and Biofilm Formation on Orthopedic Implants

The 18 h culture of *Staphylococcus* spp. on Columbia Agar (Becton-Dickinson, Warszawa, Poland) (37 °C) was used to prepare a bacterial suspension of 1 McFarland standard (Densitometer Densimat; BioMerieux, Warszawa, Poland) in liquid TSB (Biomaxima, Lublin, Poland) medium with the addition of the peptide at a suitable concentration (subinhibitory, equivalent to MIC, a multiple of MIC). The biomaterials were introduced to the obtained suspension and were incubated for 4 h at 37 °C. Then, they were rinsed three times in a PBS solution and shaken (Vortex; BioSan, Poland) in 1 mL of 0.5% saponin (Sigma-Aldrich, Poznań, Poland) solution. The obtained bacterial suspension was cultured quantitatively on Columbia Agar with a 5% addition of sheep blood (Becton-Dickinson, Warszawa, Poland). After incubation (24 h, 37 °C), the grown bacterial colonies were counted, and the number of colony-forming units per milliliter of the suspension (cfu/mL) was determined.

A bacterial suspension without the addition of the peptide was used as a control sample.

### 4.9. Effect of Camel Peptide on Mature Biofilm on Orthopedic Implants

To obtain biofilm, the biomaterials were placed in a 1 McFarland standard (Densitometer Densimat; BioMerieux, Warszawa, Poland) bacterial suspension in liquid TSB (Biomaxima, Lublin, Poland) medium and incubated (24 h/37 °C). Then, the screws were transferred to test tubes containing a fresh TSB (Biomaxima, Lublin, Poland) medium with the addition of the peptide at a suitable concentration (subinhibitory, equivalent to MIC, multiple of MIC). The medium was incubated for another 24 h. Then, the biomaterials were rinsed three times in a PBS solution and shaken (Vortex; BioSan, Poland) in 1 mL of 0.5% saponin (Sigma-Aldrich, Poznań, Poland) solution. The obtained suspension was cultured quantitatively on Columbia Agar with a 5% addition of sheep blood (Becton-Dickinson, Warszawa, Poland). The plates were incubated (24 h, 37 °C), and then the grown bacterial colonies were counted and the number of colony-forming units per 1 mL of solution was estimated.

### 4.10. Dynamics of Biofilm Formation in the Presence of Camel Peptide

The 18 h culture of the strains was used to prepare a bacterial suspension in liquid TSB (Biomaxima, Lublin, Poland) medium (1 McFarland standard). The antibiotic was added to the suspension to obtain concentrations corresponding to subinhibitory values, MIC values and multiples of MIC. After the insertion of biomaterials, the suspension was incubated for up to 72 h, with quantitative cultures made in the 2nd, 4th, 8th, 24th, 48th and 72nd hour of incubation.

The bacterial suspension in the TSB (Biomaxima, Lublin, Poland) medium without the addition of the peptide served as a control sample for the purposes of assessing the effect of Camel peptide on the speed of biofilm formation.

### 4.11. Confocal Microscopy

Confocal microscopy was used to assess the metabolic viability of the strains in the biofilm under the influence of Camel peptide.

The implants were rinsed three times in a sterile PBS solution and were introduced into a LIVE⁄DEAD (Life Technologies, Carlsbad, CA, USA) mixture containing Syto 9 and propidium iodide (PI) prepared directly before staining in accordance with the manufacturer’s instructions. The staining was performed for 30 min at room temperature. After rinsing, the biomaterials were assessed using a confocal microscope (Zeiss Cell Observer SD—Carl Zeiss Microscopy GmbH, Jena, Germany) equipped with 10× dry objective (NA 0.3) and EMCCD Andor iXon3 885 camera. Syto 9 fluorescence was recorded using a 488 nm laser line and FE01—520/35 emission filter. PI was excited with a 561 nm laser line, and its emission was collected using a BP 629/62 filter. The surface of the implants was imaged in a reflection mode with the 488 nm laser line. The acquisition was performed in the sequential mode, and the implant area was imaged in the tile mode. Cells showing green fluorescence were regarded as live, while cells with red fluorescence were determined as dead.

### 4.12. Statistical Calculations

The analysis comparing the level of quantitative variables between the strains was performed using the Fisher test and Mann–Whitney test. The analyses were performed using the statistical package R for Windows (version 3.2.1).

## 5. Conclusions

Antimicrobial peptides have been investigated by numerous research centers in terms of their efficacy and possible application in the treatment of various infections. The presented research results indicate that the peptide exhibits very good antimicrobial properties against the analyzed strains. The results of our study on the Camel peptide offer a possibility of its application in the treatment of orthopedic infections caused by *S. epidermidis* and *S. haemolyticus* and, what is more important and worth stressing, in the prevention of such infections.

## Figures and Tables

**Table 1:** Methicillin resistance among *S. epidermidis* and *S. haemolyticus* strains.

Species	Sensitive	Resistant
All strains	38%	62%
*S. epidermidis* (n = 61)	64%	36%
*S. haemolyticus* (n = 60)	12%	88%

**Table 2:** Minimal inhibitory concentration (MIC) and minimal biofilm eradication concentration (MBEC) of Camel peptide with respect to the analyzed strains.

Species	MIC µg/mL (Median)	MBEC µg/mL (Median)
*S. epidermidis*	2–32 (8)	4–>250 (32)
*S. haemolyticus*	2–32 (4)	4–128 (16)

## Data Availability

All data generated or analyzed during this study are included in this published article and Supplementary Materials.

## Supplementary Materials

The following supporting information can be downloaded at https://www.mdpi.com/article/10.3390/antibiotics12121671/s1, Table S1: Methicillin resistance among *S. epidermidis* and *S. haemolyticus* strains.; Table S2: MIC and MBEC of Camel peptide with respect to *S. epidermidis* strains.; Table S3: MIC and MBEC of Camel peptide with respect to *S. haemolyticus* strains.; Table S4: Quantitative evaluation (cfu/mL) of the ability to form biofilm for analyzed strains.
