# Supplementary material for: Effect of Camel Peptide on the Biofilm of Staphylococcus epidermidis and Staphylococcus haemolyticus Formed on Orthopedic Implants
Source: Antibiotics (Basel). 2023 Nov 28;12(12):1671. doi: 10.3390/antibiotics12121671 (PMC10740474; doi:10.3390/antibiotics12121671)
Supplement: Supplementary file 1 [file antibiotics-12-01671-s001.zip › antibiotics-2733283-supplementary.pdf]

antibiotics

MDPI

Supplementary Materials

Effect of Camel Peptide on the Biofilm of *Staphylococcus epidermidis* and *Staphylococcus haemolyticus* Formed on Orthopedic Implants

Joanna Nowicka [1,*], Adriana Janczura [1,*], Magdalena Pajączkowska [1], Grzegorz Chodaczek [2], Patrycja Szymczyk-Ziółkowska [3], Urszula Walczuk [1] and Grażyna Gościniak [1]

[1] Department of Microbiology, Faculty of Medicine, Medical University, 50-368 Wrocław, Poland; magdalena.pajaczkowska@umw.edu.pl (M.P.); urszula.walczuk@umw.edu.pl (U.W.); grazyna.gosciniak@umw.edu.pl (G.G.)
[2] Bioimaging Laboratory, Łukasiewicz Research Network—PORT Polish Center for Technology Development, 54-066 Wrocław, Poland; grzegorz.chodaczek@port.lukasiewicz.gov.pl
[3] Centre for Advanced Manufacturing Technologies (CAMT/FPC), Faculty of Mechanical Engineering, Wroclaw University of Science and Technology, Łukasiewicza 5, 50-371 Wroclaw, Poland; patrycja.e.szymczyk@pwr.edu.pl
* Correspondence: joanna.nowicka@umw.edu.pl (J.N.); adriana.janczura@umw.edu.pl (A.J.)

Table S1. Methicillin resistance among *S. epidermidis* and *S. haemolyticus* strains.

Strain number	*S. epidermidis*	*S. haemolyticus*
1	Resistant	Resistant
2	Sensitive	Sensitive
3	Sensitive	Sensitive
4	Sensitive	Resistant
5	Sensitive	Resistant
6	Sensitive	Resistant
7	Resistant	Resistant
8	Sensitive	Resistant
9	Resistant	Resistant
10	Resistant	Resistant
11	Resistant	Resistant
12	Sensitive	Resistant
13	Sensitive	Resistant
14	Resistant	Sensitive
15	Resistant	Resistant
16	Sensitive	Resistant
17	Resistant	Resistant
18	Resistant	Resistant
19	Sensitive	Resistant
20	Sensitive	Sensitive
21	Sensitive	Resistant
22	Sensitive	Resistant
23	Sensitive	Resistant
24	Sensitive	Resistant
25	Resistant	Resistant
26	Resistant	Resistant
27	Resistant	Resistant

28	Sensitive	Resistant
29	Sensitive	Resistant
30	Sensitive	Resistant
31	Resistant	Resistant
32	Resistant	Resistant
33	Sensitive	Resistant
34	Resistant	Resistant
35	Sensitive	Resistant
36	Sensitive	Resistant
37	Sensitive	Resistant
38	Resistant	Resistant
39	Resistant	Resistant
40	Sensitive	Resistant
41	Sensitive	Resistant
42	Sensitive	Resistant
43	Sensitive	Resistant
44	Resistant	Resistant
45	Sensitive	Sensitive
46	Resistant	Resistant
47	Sensitive	Resistant
48	Sensitive	Resistant
49	Sensitive	Resistant
50	Sensitive	Resistant
51	Sensitive	Resistant
52	Sensitive	Resistant
53	Sensitive	Resistant
54	Resistant	Resistant
55	Resistant	Resistant
56	Sensitive	Sensitive
57	Sensitive	Sensitive
58	Sensitive	Resistant
59	Resistant	Resistant
60	Sensitive	Resistant
61	Sensitive	

Table S2. MIC and MBEC of Camel peptide in respect of *S. epidermidis* strains.

Strain number	MIC (µg/mL)	MBEC (µg/mL)
1	8	256
2	8	64
3	8	32
4	32	> 256
5	32	> 256
6	32	256
7	16	64
8	16	256
9	16	> 256
10	8	128
11	8	32
12	2	32

13	8	16
14	2	32
15	8	32
16	8	256
17	16	256
18	8	256
19	4	16
20	2	32
21	2	16
22	8	32
23	8	32
24	8	64
25	8	32
26	4	4
27	8	16
28	8	16
29	8	32
30	8	32
31	16	32
32	8	32
33	16	64
34	16	64
35	16	256
36	8	32
37	8	32
38	8	125
39	16	32
40	16	16
41	16	256
42	16	64
43	8	32
44	16	32
45	8	32
46	8	64
47	8	128
48	8	256
49	8	256
50	8	128
51	4	16
52	4	256
53	4	256
54	16	256
55	8	32
56	32	> 256
57	16	128
58	8	32
59	32	64
60	4	16
61	4	8

Table S3. MIC and MBEC of Camel peptide in respect of *S. haemolyticus* strains.

Strain number	MIC (μg/mL)	MBEC (μg/mL)
1	4	16
2	4	16
3	4	32
4	2	8
5	8	64
6	4	16
7	4	16
8	2	32
9	2	16
10	2	8
11	8	128
12	4	16
13	4	16
14	8	128
15	4	32
16	2	16
17	4	16
18	4	16
19	4	8
20	2	16
21	4	64
22	4	16
23	4	16
24	4	16
25	4	16
26	4	16
27	4	16
28	4	8
29	2	64
30	4	16
31	2	16
32	2	16
33	4	64
34	4	16
35	2	16
36	8	128
37	8	128
38	16	16
39	16	128
40	32	128
41	16	32
42	16	128
43	16	64
44	2	64
45	2	16
46	2	16

47	4	16
48	2	4
49	2	16
50	8	32
51	4	8
52	4	8
53	2	128
54	16	128
55	2	16
56	4	32
57	4	8
58	4	16
59	2	8
60	2	8

Table S4. Quantitative evaluation (cfu/mL) of the ability to form biofilm for analyzed strains.

Strain number	*S. epidermidis*	*S. haemolyticus*
1	1,8x10^{7*}	3,4x10^{6*}
2	2x10^{5}	1,95x10^{6}
3	6,7x10^{7}	1,1x10^{7}
4	6,4x10^{7}	2,88x10^{6}
5	3,9x10^{7}	1,21x10^{6}
6	1,8x10^{7}	1,8x10^{5}
7	5,7x10^{6}	1,0x10^{6}
8	4,0x10^{7}	2,3x10^{6}
9	1,0x10^{5}	1,8x10^{5}
10	1,1x10^{8}	2,6x10^{6}
11	6,58x10^{6}	4,5x10^{6}
12	1,3x10^{5}	1,37x10^{7}
13	1,0x10^{7}	3,2x10^{6}
14	3,0x10^{6}	3,27x10^{6}
15	4,8x10^{5}	1,84x10^{6}
16	1,87x10^{7}	1,75x10^{5}
17	3,0x10^{7}	1,2x10^{5}
18	1,75x10^{7}	4,1x10^{5}
19	5,72x10^{7}	2,12x10^{6}
20	5,9x10^{7}	3,2x10^{4}
21	2,8x10^{6}	2,35x10^{7}
22	8,5x10^{6}	1,39x10^{7}
23	6,8x10^{6}	6,68x10^{6}
24	7,1x10^{6}	2,5x10^{6}
25	9,0x10^{5}	7,58x10^{6}
26	2,68x10^{7}	1,02x10^{7}
27	1,65x10^{7}	7x10^{5}
28	1,36x10^{7}	1,42x10^{7}
29	2,96x10^{7}	6,4x10^{6}
30	9,95x10^{6}	4,3x10^{6}
31	1,09x10^{7}	3,0x10^{6}
32	1,48x10^{7}	44,0x10^{6}

33	$2,1x10^7$	$6,8x10^6$
34	$5,29x10^7$	$8,9x10^6$
35	$1,32x10^9$	$2,5x10^6$
36	$1,59x10^7$	$4,9x10^6$
37	$1,8x10^6$	$1,5x10^6$
38	$6,23x10^6$	$5,16x10^6$
39	$5,0x10^5$	$2,5x10^7$
40	$1,1x10^7$	$6,24x10^6$
41	$1,9x10^6$	$2,62x10^6$
42	$2,38x10^6$	$2,6x10^6$
43	$4,41x10^6$	$7,5x10^6$
44	$1,74x10^6$	$3,4x10^7$
45	$4,14x10^6$	$2,67x10^6$
46	$7,0x10^5$	$5,1x10^5$
47	$4,1x10^5$	$3,2x10^6$
48	$3,4x10^6$	$4,0x10^4$
49	$2,91x10^6$	$3,2x10^7$
50	$2,0x10^6$	$6,8x10^5$
51	$1,32x10^6$	$6,6x10^5$
52	$2,5x10^7$	$2,68x10^6$
53	$2,49x10^7$	$2,3x10^6$
54	$6,2x10^7$	$1,1x10^6$
55	$3,72x10^6$	$3,8x10^6$
56	$7,92x10^7$	$2,3x10^6$
57	$2,43x10^6$	$2,3x10^6$
58	$7,86x10^9$	$1,2x10^7$
59	$6,87x10^7$	$1,28x10^6$
60	$3,24x10^6$	$3,12x10^5$
61	$1,85x10^5$	
RP 62A	$7,5x10^7$	
